# Supplementary material for: Postoperative Complications Are Associated with Long-Term Changes in the Gut Microbiota Following Colorectal Cancer Surgery
Source: Life (Basel). 2021 Mar 16;11(3):246. doi: 10.3390/life11030246 (PMC8002283; doi:10.3390/life11030246)
Supplement: Supplementary file 1 [file life-11-00246-s001.pdf]

life MDPI

Supplementary Material

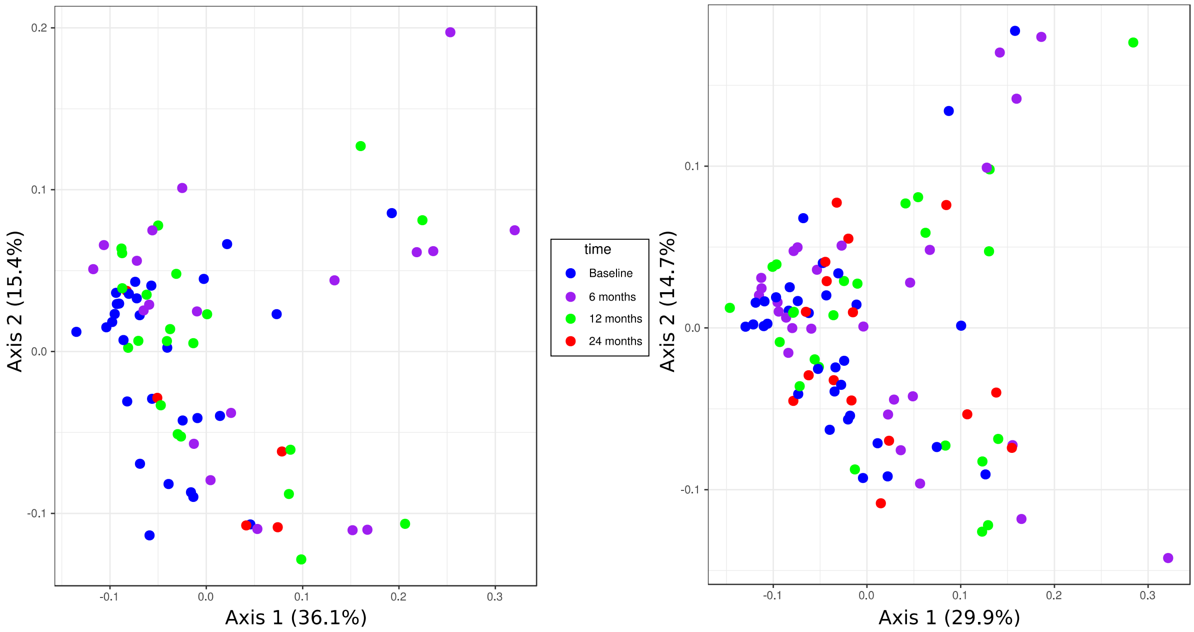

Figure S1. Microbial composition visualised by principal coordinate analysis (PCoA). PCoA on the left side presents the data of patients within the group with complications and on the right side the data of the non-complication group. Different colours correspond to the distinct timepoints: blue represents baseline, purple represents 6 months, green represents 12 months and red represents 24 months. Postoperative complications also affect the structure of the microbiome 6 months after surgery in patients with complications (R2=0.09, p-value =0.006). In the non-complication group, no structural differences were detected.

Table S1. Differentially abundant RSV at baseline between patient with complication and patient without complication. Test was performed using Deseq 2 and a positive log2 (Fold change) indicates an enrichment in the group with complications.

RSV	LOG2 FOLDCHANGE	PADJ	TAXONOMY
**RSV4**	24,3388578	1,4933E–14	Verrucomicrobia;Verrucomicrobiae;Verrucomicrobiales; Akkermansiaceae;Akkermansia;muciniphila
**RSV7**	–10,386	0,00173794	Firmicutes;Negativicutes;Selenomonadales; Acidaminococcaceae;Phascolarctobacterium;faecium
**RSV122**	25,0570292	3,5616E–15	Bacteroidetes;Bacteroidia;Bacteroidales; Muribaculaceae;unclassified
**RSV177**	24,9228975	3,5616E–15	Firmicutes;Clostridia;Clostridiales;Ruminococcaceae; Ruminococcaceae_UCG–014;unclassified
**RSV247**	–23,524721	2,6589E–17	Proteobacteria;Gammaproteobacteria;Betaproteobacteriales; Burkholderiaceae;Sutterella;unclassified
**RSV283**	23,6306827	7,6214E–14	Bacteroidetes;Bacteroidia;Bacteroidales;Prevotellaceae; Prevotella_9;unclassified
**RSV356**	23,255365	1,862E–13	Bacteroidetes;Bacteroidia;Bacteroidales; Muribaculaceae;unclassified
**RSV366**	23,1530754	2,186E–13	Bacteroidetes;Bacteroidia;Bacteroidales; Marinifilaceae;Butyricimonas;unclassified
**RSV424**	–23,983283	3,5947E–14	Firmicutes;Clostridia;Clostridiales; Ruminococcaceae;unclassified

Table S2. Food group factor loadings for the "western", "fruit & vegetable" and "bread & butter" dietary pattern, identified using PCA.

Food groups	Western	Bread and Butter	Fruit and Vegetable
Potatoes	0.34182	0.09982	0.08548
Vegetables	0.02552	0.06753	**0.77370**
Legumes	0.24123	–0.02070	0.15801
Fruits	–0.09217	0.04007	**0.44247**
Dairy Products	0.15488	0.02846	0.04978
Pasta, Rice, and Other gGrain	0.24562	0.04302	–0.07426
Bread	0.07248	**0.86524**	–0.01947
Grains	0.16318	0.00263	–0.05091
Red meat	**0.56690**	0.10920	0.09055
Poultry	**0.42827**	–0.01106	0.06643
Processed Meat	**0.63896**	0.16371	–0.03092
Fish and Shellfish	0.16942	0.11707	0.16117
Eggs and Egg Products	0.24525	0.08454	0.05436
Vegetable Oil	0.14557	–0.06443	**0.57565**
Butter	0.01076	**0.76734**	0.04804
Margarine	0.16780	0.27529	0.06402
Frying Fat	0.08789	0.17593	0.27700
Sweets	0.24228	0.17268	0.01400
Cakes	0.26255	0.04549	0.03868
Non–Alcohol	0.04391	0.02291	0.19266
Wine	0.03815	0.04914	–0.03866
Other Alcohol	0.12445	0.03436	–0.06308
Sauce and Condiments	0.28162	–0.02826	0.11408
Soups	0.19334	0.01960	0.11951
Soy	0.01516	–0.04436	0.17510

Factor loadings greater than |0.40| are shown in bold
PCA=Principal component analysis
